# Interactions between dopamine transporter and N-methyl-D-aspartate receptor-related amino acids on cognitive impairments in schizophrenia

**DOI:** 10.1192/j.eurpsy.2023.1058

**Published:** 2023-07-19

**Authors:** Y.-H. Chou

**Affiliations:** Tapei Veterans General Hospital, Taipei, Taiwan, Province of China

## Abstract

**Introduction:**

Cognitive impairments, the main determinants of functional outcomes in schizophrenia, had limited treatment responses and need a better understanding of the mechanisms. Dysfunctions of the dopamine system and N-methyl-D-aspartate receptor (NMDAR), the primary pathophysiologies of schizophrenia, may impair cognition.

**Objectives:**

This study explored the effects and interactions of striatal dopamine transporter (DAT) and plasma NMDAR-related amino acids on cognitive impairments in schizophrenia.

**Methods:**

We recruited 36 schizophrenia patients and 36 age- and sex-matched healthy controls (HC). All participants underwent cognitive assessments of attention, memory, and executive function. Single-photon emission computed tomography with 99mTc-TRODAT and ultra-performance liquid chromatography were applied to determine DAT availability and plasma concentrations of eight amino acids, respectively.

**Results:**

Compared with HC, schizophrenia patients had lower cognitive performance, higher methionine concentrations, decreased concentrations of glutamic acid, cysteine, aspartic acid, arginine, the ratio of glutamic acid to gamma-aminobutyric acid (Glu/GABA), and DAT availability in the left caudate nucleus (CN) and putamen. Regarding memory scores, Glu/GABA and the DAT availability in left CN and putamen exhibited positive relationships, while methionine concentrations showed negative associations in all participants. The DAT availability in left CN mediated the methionine-memory relationship. An exploratory backward stepwise regression analysis for the four biological markers associated with memory indicated that DAT availability in left CN and Glu/GABA remained in the final model.

**Conclusions:**

This study demonstrated the interactions of striatal DAT and NMDAR-related amino acids on cognitive impairments in schizophrenia. Future studies to comprehensively evaluate their complex interactions and treatment implications are warranted.

**Disclosure of Interest:**

None Declared

